# Balloon atrial septostomy through internal jugular vein in a 45-day-old child with transposition of great arteries

**DOI:** 10.4103/0974-2069.64363

**Published:** 2010

**Authors:** Sumanta S Padhi, Kinjal D Bakshi, Avinash P Londhe

**Affiliations:** Department of Pediatric Cardiology, Innova Children's Heart Hospital, Secunderabad, India; 1 Department of Anesthesiology, Innova Children's Heart Hospital, Secunderabad, India

**Keywords:** Balloon atrial septostomy, internal jugular vein, transposition of great arteries

## Abstract

Balloon atrial septostomy is a common palliative procedure in D-transposition of great arteries. It is technically easy before 2-3 weeks of age when the septum primum is thin. Femoral vein or umbilical vein, when available, is the common access used for this procedure. In situations when these accesses are not available or in case of inferior vena cava interruption, trans-hepatic access is used. Internal jugular vein (IJV) access is not used as it is difficult to enter the left atrium through this route. We describe a case of successful Balloon atrial septostomy done through IJV in a 45-day-old child with emphasis on the technique, hardware and precautions necessary during the procedure.

## INTRODUCTION

Balloon atrial septostomy (BAS) was described by Rashkind and Miller in 1966 as a palliative procedure for D-transposition of great arteries (D-TGA).[1] The procedure is still commonly performed in developing countries like India where complete repair during the neonatal period is not widely available. For a successful BAS, a thin septum primum is essential. Hence the procedure yields best result if performed before 2 to 3 weeks. Femoral vein or umbilical vein, whenever available, is the access of choice for the procedure. In cases where either of the access is not available like thrombosed vein due to previous use, or interrupted inferior vena cava (IVC), trans-hepatic approach is used.[2] IJV is not used as it is difficult to enter LA through this route and the perceived risk of injuring the sinoatrial node (SA node). Infants older than 1 to 2 months (due to thick septum primum) and those with interrupted IVC pose technical challenges for performing BAS.[3]

## CASE REPORT

A 45-day-old child was received in the emergency room with severe cyanosis (Pulse oxymetry saturation of 20-30%). He was being treated for 'hypercyanotic spell' at a local hospital and was referred to us as there was no improvement in his clinical condition. On evaluation, the child was found to have D-TGA, intact ventricular septum, mild dynamic left ventricular obstruction, restrictive fossa ovalis atrial septal defect (FO ASD) with bidirectional flow. There was no patent ductus arteriosus. The left ventricular (LV) shape was maintained and its thickness appeared to be preserved. The septum primum was relatively thin.

Because of financial issues, atrial switch operation (ASO) was not possible. As the fossa ovalis region was relatively thin, it was decided that BAS can be attempted for stabilization despite the age of child.

**Figure 1:**
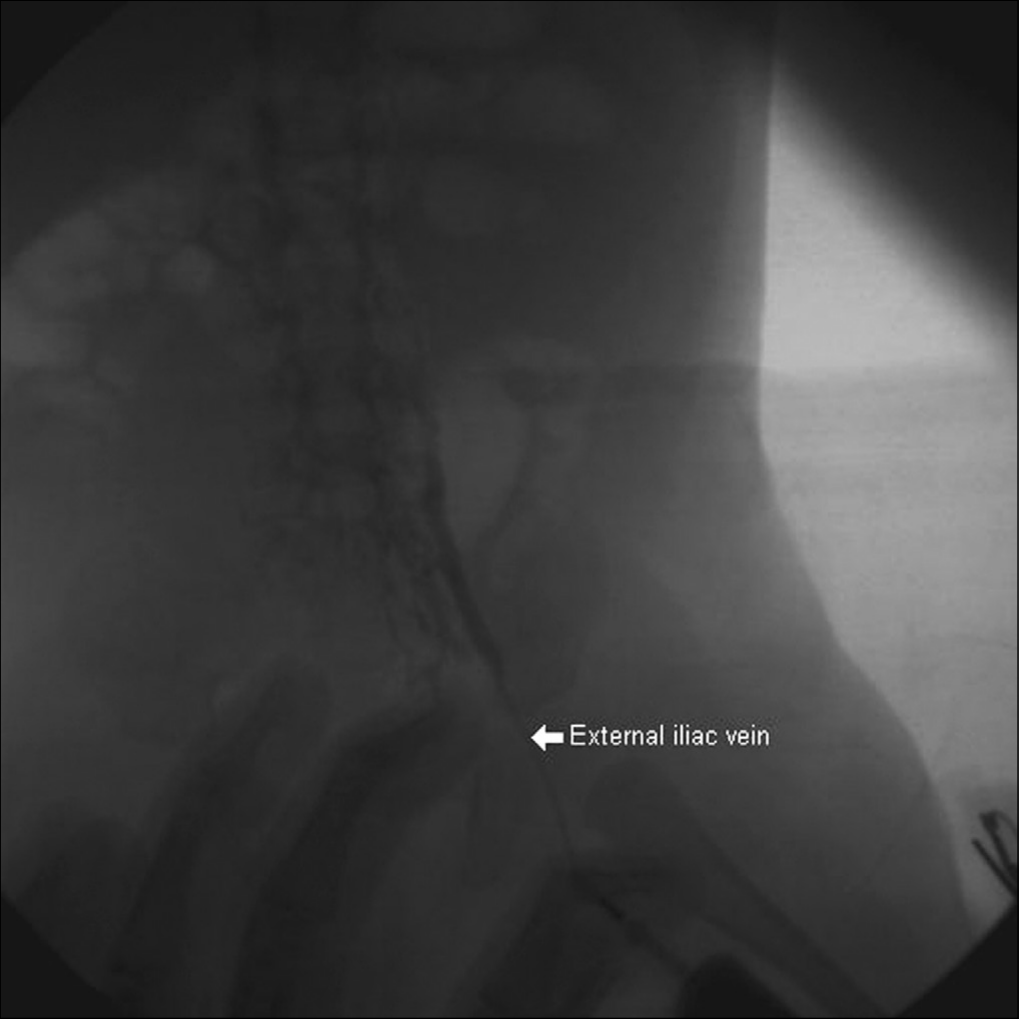
Left femoral venogram showing blocked external iliac vein. The vein that reforms distally passes along the right border of vertebra suggesting blocked lower end of inferior vena cava also

**Figure 2:**
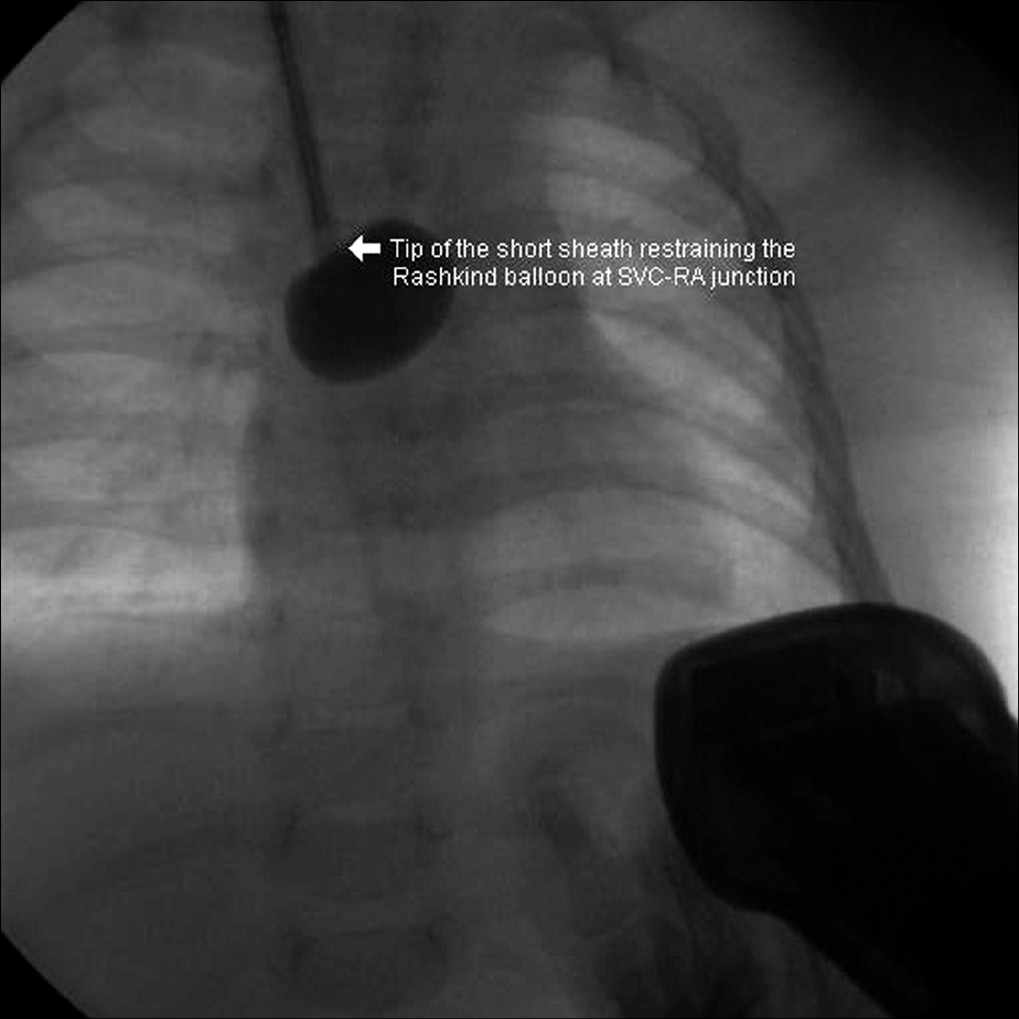
Short sheath through IJV, the tip of which is restraining the Rashkind balloon at SVC-RA junction

**Figure 3:**
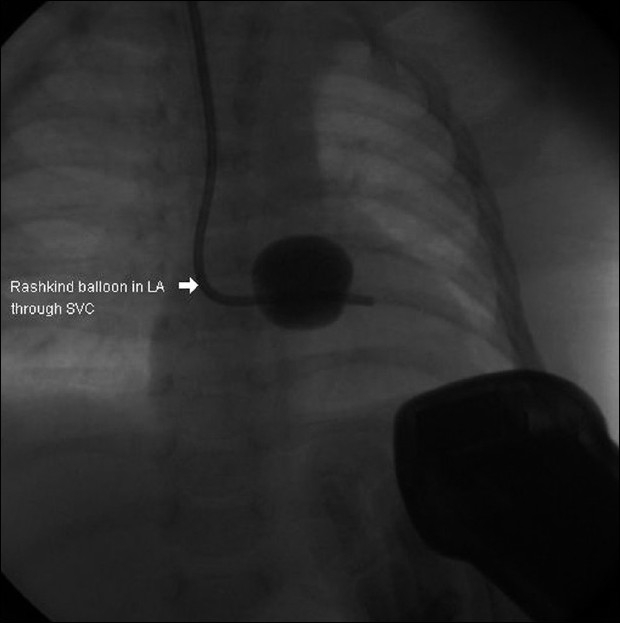
Rashkind balloon in LA, which was passed through the PFO after pre-shaping it with pacemaker lead stylet

The patient was taken for BAS after intubation and mechanical ventilation. However, attempts to get the femoral venous access on either side were not successful. A left femoral venogram showed external iliac vein occlusion and evidence of occlusion of lower end of IVC [Figure 1]. Hence the IJV access was taken and a 7F sheath was introduced. Care was taken to keep the tip of the sheath beyond the superior vena cava – right atrial junction, so that injury to the SA node could be avoided during the 'pull' of the catheter [Figure 2]. The position of the tip was checked prior to performing the procedure. Since the Rashkind balloon could not be negotiated to LA by standard maneuver through the FO ASD, the balloon was pre-shaped to 90° angulation by inserting a pacemaker lead stylet [Figure 3]. Once the balloon entered the LA, the stylet was removed from the balloon and septostomy could be done similar to that is done through IVC route. However, even after three attempts, a sufficiently large ASD with good flap could not be made, even though there was increase in the saturations from about 30% to 50–55%.

**Figure 4:**
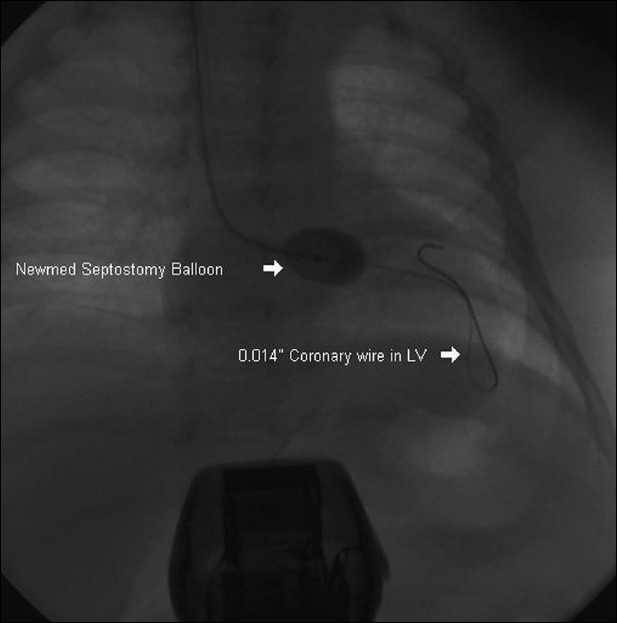
NuMED atrioseptostomy catheter in the left atrium passed over a 0.014” coronary wire, which is parked in left ventricle

Subsequently, the ASD was crossed with a 0.014” coronary wire with support of 4F right coronary artery catheter. The distal end of the wire was parked in the LV. Over the wire a NuMED Z-5™ SPT 002–9.5 mm atrioseptostomy catheter (NuMED, Inc.) was introduced and BAS was performed [Figure 4]. A 5.1 mm ASD, with good bidirectional laminar flow could be created after the procedure. The saturation improved up to 75 to 78%.

The procedure could be completed without any complications. The child could be extubated same day. The corrective surgery could not be done in the same admission due to financial reason. At 10-day follow-up after the procedure the child is still saturating 72% and waiting for the corrective surgery.

## DISCUSSION

One of the main causes of early mortality in case of D-TGA is hypoxia secondary to restrictive ASD.[4,5] BAS is one of the life-saving palliative procedures that can be performed in catheterization laboratory or even intensive care unit under transthoracic echocardiography.[6] The femoral vein or umbilical vein, whenever available, is used as the access for the procedure. In situations where these accesses are not available e.g. thrombosed veins due to previous use, or in case of IVC interruption, BAS can be performed through transhepatic approach.[2,7,8] IJV access is not considered due to difficulties mentioned earlier. In our case both the external iliac veins were thrombosed during treatment in the previous hospitalization. The transhepatic route was not accessed due to the reported rate of intra-peritoneal bleed as high as 4.5% even in experienced hands.[9] At 45 days of life, blade atrial septostomy would have been a better option than balloon septostomy, however it was not considered, as it was felt that operator would not get a proper angulation to position the blade through the IJV route. We could enter the LA successfully with a Rashkind balloon with technique described above. However we could not achieve the desired result, even though the procedure could be done without complication. The subsequent option was to use an over-the-wire balloon like the NuMED Z-5™ SPT 002–9.5 mm atrioseptostomy catheter (NuMED, Inc.). The procedure could be done successfully and a satisfactory result could be achieved.

The aim of reporting this case is to emphasize that, in difficult situations where femoral vein or umbilical vein is not accessible or in case of IVC interruption, successful BAS could be done through the IJV route by pre-shaping the Rashkind balloon or using over-the-wire septostomy balloon (e.g. NuMED Z-5™ atrioseptostomy catheter). However careful echocardiographic evaluation of interatrial septum is needed and the success of the procedure depends on the presence of thin septum primum. During the procedure care should be taken to prevent injury to the SA node.
